# Determinants and Effects of Voice Disorders among Secondary School Teachers in Peninsular Malaysia Using a Validated Malay Version of VHI-10

**DOI:** 10.1371/journal.pone.0141963

**Published:** 2015-11-05

**Authors:** Foong Ming Moy, Victor Chee Wai Hoe, Noran Naqiah Hairi, Anne Hin Yee Chu, Awang Bulgiba, David Koh

**Affiliations:** 1 Julius Centre University of Malaya, Department of Social & Preventive Medicine, Faculty of Medicine, University of Malaya, Kuala Lumpur, Malaysia; 2 Centre for Occupational and Environmental Health-UM, Department of Social & Preventive Medicine, Faculty of Medicine, University of Malaya, Kuala Lumpur, Malaysia; 3 PAPRSB Institute of Health Sciences, Universiti Brunei Darussalam, Brunei, Brunei Darussalam; 4 Saw Swee Hock School of Public Health, National University of Singapore, Singapore, Singapore; Ulm University, GERMANY

## Abstract

**Objectives:**

To establish the prevalence of voice disorder using the Malay-Voice Handicap Index 10 (Malay-VHI-10) and to study the determinants, quality of life, depression, anxiety and stress associated with voice disorder among secondary school teachers in Peninsular Malaysia.

**Methods:**

This study was divided into two phases. Phase I tested the reliability of the Malay-VHI-10 while Phase II was a cross-sectional study with two-stage sampling. In Phase II, a self-administered questionnaire was used to collect socio-demographic and teaching characteristics, depression, anxiety and stress scale (Malay version of DASS-21); and health-related quality of life (Malay version of SF12-v2). Complex sample analysis was conducted using multivariate Poisson regression with robust variance.

**Results:**

In Phase I, the Spearman correlation coefficient and Cronbach alpha for total VHI-10 score was 0.72 (p < 0.001) and 0.77 respectively; showing good correlation and internal consistency. The ICCs ranged from 0.65 to 0.78 showing fair to good reliability and demonstrating the subscales to be reliable and stable. A total of 6039 teachers participated in Phase II. They were primarily Malays, females, married, had completed tertiary education and aged between 30 to 50 years. A total of 10.4% (95% CI 7.1, 14.9) of the teachers had voice disorder (VHI-10 score > 11). Compared to Malays, a greater proportion of ethnic Chinese teachers reported voice disorder while ethnic Indian teachers were less likely to report this problem. There was a higher prevalence ratio (PR) of voice disorder among single or divorced/widowed teachers. Teachers with voice disorder were more likely to report higher rates of absenteeism (PR: 1.70, 95% CI 1.33, 2.19), lower quality of life with lower SF12-v2 physical (0.98, 95% CI 0.96, 0.99) and mental (0.97, 95% CI 0.96, 0.98) component summary scales; and higher anxiety levels (1.04, 95% CI 1.02, 1.06).

**Conclusions:**

The Malay-VHI-10 is valid and reliable. Voice disorder was associated with increased absenteeism, marginally associated with reduced health-related quality of life as well as increased anxiety among teachers.

## Introduction

Teachers form a significant proportion of the global workforce and this is also true in Malaysia. According to the Ministry of Education, Malaysia, there were a total of 408,764 teachers in 2010 [1] which made up 3.9% of the country’s workforce (10.4 million). Teaching is a profession that is vocally demanding where one’s voice is used as his/her primary tool. Excessive use or abuse of one’s voice at work can lead to voice disorders [2].

Teachers place heavy demands on their voice, often instructing for many hours in acoustically challenging environments such as noisy classrooms, without adequate time for the vocal cords to rest and recover. Noisy classrooms force teachers to increase vocal loudness and this predisposes them to voice disorders. Voice disorders among school teachers lead to adverse outcomes such as reduction in quality of life, decreased work performance, increased absenteeism, and restriction of social activities or interactions [3,4]. Teachers may even be forced to end their careers early because of vocal difficulties [4].

The prevalence of voice disorders varies depending on the methodology used in studies (e.g. objective measures such as laryngoscopy or self-reported voice disorders using questionnaires), definitions and recall period [5]. Prevalence estimates of voice disorders among Brazilian teachers ranged from 15% to 89% [4]. Another epidemiological study of voice disorders among teachers in the United States showed 11% of them reported suffering from current voice disorder, and 58% experienced voice disorder during their lifetime [6]. Another review reported prevalence rates of voice disorders among teachers ranging between 4.4% and 90% [7]. There is a paucity of published reports on voice disorders among teachers in the South East Asian region. -To date, only one small study from Singapore reported voice disorders among 214 primary school teachers with a point prevalence of 13.1% and career prevalence of 25.4% [8].

Risk factors for voice disorders among teachers include long working hours, excessive number of students per classroom, environmental noise and inappropriate classroom facilities [9]. The female gender, age and duration of employment in the profession are also parameters that may contribute to voice disorders among teachers [2,10–12].

To understand the problem, a reliable tool in evaluating voice disorders is essential. Voice disorders can be evaluated with the use of objective tools as well as perceptual approaches. However it has been suggested that voice disorders cannot be fully measured by objective tools alone as the outcome involves the assessment of issues such as how the voice problem or the level of disability experienced by the individual [13]. Perceptual measurement of voice disorder involves assessing an individual’s ability to speak using his or her voice under normal conditions in daily work and social settings [13,14].

A commonly used tool for evaluating voice disorder is the Voice Handicap Index (VHI) developed by Jacobson et al. in 1997 [15]. VHI is a psychometrically sound instrument which is widely used and accepted for both clinical and research purposes [16]. It consists of 30 items across three domains: functional, emotional and physical aspects of the voice. This tool assesses individual’s perception of vocal difficulties. The functional domain explores the “impact of a person’s voice disorder on his or her daily activities”. The emotional domain depicts the individual’s “affective responses to the voice disorder”, while the physical domain describes the individual’s “self-perceptions of laryngeal discomfort and the voice output characteristics” [15].

The original 30-item VHI was considered lengthy and burdensome for respondents. Therefore, an abbreviated version of the VHI consisting of 10 items (VHI-10) was developed by Rosen et al [16]. From the original 30-item VHI, ten of the most robust items were extracted to form the VHI-10 and tested on 100 patients with voice disorders and 159 control individuals. Analysis of the test score comparisons between the two versions revealed a high correlation (r = 0.98), representing a comparable robustness of the VHI-10 with the original version (VHI). The VHI-10 may be a more robust instrument than the 30-item VHI where the VHI-10 scores were consistently higher than the expected value (33%) for a variety of voice disorders categories [16]. The VHI-10 has been adapted for various languages (e.g., Chinese, Hebrew, Spanish) showing good reliability and validity [17–19]; but it has never been documented in the Malay language.

This study aimed to 1) translate the VHI-10 into the Malay language version (Malay-VHI-10) and to assess its test-retest reliability among teachers; 2) establish the prevalence of voice disorder among secondary school teachers using the Malay-VHI-10 and 3) study the determinants, quality of life, depression, anxiety and stress associated with voice disorder.

## Materials and Methods

This study was divided into two phases. Phase I was a cross sectional study carried out in January 2013 to test the reliability of the Malay-VHI-10 and Phase II (carried out from February 2013 to June 2014) was also a cross-sectional study assessing the prevalence of voice disorder and its association with socio-demographic characteristics, teaching characteristics, health-related quality of life, depression, anxiety and stress among secondary school teachers.

### Phase I: Translation and test-retest of the Malay-VHI-10

We only carried out the reliability test (using a test-retest method) on the Malay-VHI-10 as the VHI-10 has been proven to be valid across a variety of cultures and languages [17–21]. The VHI-10 developed by Rosen et al [16] consists of 10 items which requires the participant to rate each item using a five-point Likert scale (0: never, 1: almost never, 2: sometimes, 3: almost always, 4: always). Items 1 to 5 represent the functional subscale; items 6, 7 and 10 represent the physical subscale; and items 8 and 9 represent the emotional subscale. The total score was calculated by summing the score for each item to indicate the severity of voice disorders (maximum score = 40, minimum score = 0).

The VHI-10 was translated into the Malay language, which is the national language of Malaysia. Forward and backward translations of the questionnaire were conducted. Two professional translators translated the VHI-10 into Malay. Both translations were reviewed and reconciled to a single Malay version. The translation back to English was undertaken by another two bilingual translators blinded to the original English version. Discrepancies that arose were discussed and refined to ensure that the Malay version reflected the meaning of the original VHI-10.

Two schools in Kuala Lumpur were randomly selected and all teachers were invited to participate in the reliability testing. A total of 165 out of 242 teachers (73.1%) responded. The respondents were required to complete two sets of Malay-VHI-10 in a two-week interval. A two-week interval was selected as a period of 2 to 14 days apart for test—retest is considered adequate for the interval to be long enough to reduce the effects of memory but short enough to diminish the likelihood of systematic alterations [22].

### Phase II: Prevalence of voice disorder and its association with socio-demographic characteristics, teaching characteristics, health related quality of life, depression, anxiety and stress

#### Study design and sampling method

This was a cross-sectional study with two-stage sampling. Six out of the 12 states in Peninsular Malaysia (Penang, Kuala Lumpur, Selangor, Melaka, Terengganu and Johor) were randomly selected for the first stage. For the second stage, all the districts in each of the selected states were included and 70% of all public secondary schools from each district were randomly selected and invited to participate in the study. All teachers from the selected schools who fulfilled the inclusion criteria (teachers on permanent employment and free of mental health problems) were invited to participate. Participation by the schools and teachers were voluntary. For this paper, we will report data collected from the states of Penang, Kuala Lumpur, Selangor and Melaka as data for the other two states were not available.

#### Ethical considerations

Ethical approval was obtained (Reference Number: 950.1) from the Medical Ethics Committee of the University Malaya Medical Centre (UMMC) which governs all research involving human subjects in the Faculty of Medicine, University of Malaya. Approval was also granted by the Ministry of Education, Malaysia, the selected State Education Departments and principals of all invited schools. All participants were briefed on the study and asked to provide written informed consent prior to data collection.

#### Data collection

A validated and pre-tested questionnaire was self-administered by all participants. Information collected in the questionnaire included socio-demographic characteristics, teaching characteristics, depression, anxiety and stress scales using the Malay version of DASS21 [23], health-related quality of life using the Malay version of SF12-v2 [24,25] and the Malay-VHI-10. The study protocol has been published elsewhere [26].

Socio-demographic characteristics such as gender, race, marital status, age and education levels were collected. Teaching characteristics such as years of teaching and hours of teaching per day were recorded. Data on voice-related absenteeism and medical leave granted by medical doctors due to voice problems was also collected. Respondents were also asked if they used voice amplifiers during teaching and if they took any measures to minimise voice problems (such as reduced class size or change of teaching subjects).

#### Malay-Voice Handicap Index 10

The Malay version of Voice Handicap Index 10 (Malay-VHI-10) (Table 1) was used to assess voice disorders. It served as a tool to confirm an individual’s perception of the severity of one’s voice problem. The greater the total score, the greater the handicap relating to voice problems. According to Arffa et al [15], the normative value among participants without voice disorder was 2.83 (standard deviation = 3.93). They proposed that a VHI-10 total score >11 should be considered abnormal. A similar cut-off point to define voice disorder was also used by Sampaio et al [27]. Therefore, participants with scores greater than 11 were categorised as having voice disorders in this study.

**Table 1:** Original and Malay-VHI-10.

*My voice makes it difficult for people to hear me*.
**Suara saya menyebabkan orang lain sukar untuk mendengar apa yang saya kata**.
*People have difficulty understanding me in a noisy room*.
**Orang sukar memahami apa yang saya berkata apabila berada di dalam bilik yang bising**.
*My voice difficulties restrict my personal and social life*.
**Masalah suara saya menyusahkan kehidupan peribadi dan sosial saya**.
*I feel left out of conversation because of my voice*.
**Saya rasa tersisih daripada perbualan disebabkan suara saya**.
*My voice problem causes me to lose income*.
**Masalah suara saya menyebabkan saya kekurangan pendapatan**.
*I feel as though I have to strain to produce voice*.
**Saya merasakan saya perlu bersusah payah untuk mengeluarkan suara**.
*The clarity of my voice is unpredictable*.
**Kejelasan suara saya tidak dapat diagak**.
*My voice problem upsets me*.
**Masalah suara saya menyedihkan saya**.
*My voice makes me feel handicapped*.
**Suara saya membuatkan saya rasa kekurangan sesuatu**.
*People ask*, *‘‘What’s wrong with your voice*?*”*
**Orang selalu bertanya, “Mengapa dengan suara awak?”**

#### Statistical analysis

Data were analysed using Stata Software (Stata Corp., LP, College Station, TX), version 12.0. Test-retest reliability of the Malay-VHI-10 was assessed using Spearman’s correlation coefficients (r) on the total scores between the test-retest, and further analysed using intra-class correlation coefficient (ICC) with a 95% confidence interval (CI) to assess the reliability of the questionnaire and each subscale separately.

Complex sample analysis was performed in Phase II since two-stage sampling was used. The first stage sample was based on clusters of states. From the selected states, 70% of schools from all the districts were sampled. Weights, a multiplicative inverse of probability of selection, were applied to correct for unequal selection probabilities and non-response to produce unbiased estimates.

Univariate analyses were performed to identify associations between voice disorder with socio-demographic and teaching characteristics, physical health (SF12v2 Physical Component Summary scale (SF12v2-PCS)), mental health (SF12v2 Mental Component Summary scale (SF12v2-MCS)), depression, anxiety and stress.

The prevalence ratios (PR) were calculated instead of odds ratio (OR) as PR is more interpretable and it provides better estimate than OR [28]. Using OR in common diseases/conditions (more than 10%) in cross-sectional studies tends to result in overestimates of the strength of association. Multivariate Poisson regression with robust variance (when binomial regression models did not converge)[29] was performed to test which of the determinants were independently associated with voice disorder. Variables with p value of < 0.25 in the univariate analysis were included in the multivariate model as the use of p value of 0.05 had been shown to be too stringent, as important variables were often excluded from the model when this value was used.

## Results

### Phase I

The respondents who participated in Phase I aged between 25 and 59 years (mean 41.2 ± 8.5 years), were predominantly Malays (79.4%) and females (93.9%). There was no difference in race and age among respondents and non-respondents. More females than males participated in the study. Mean scores for total, functional, physical and emotional subscales obtained from the Malay-VHI-10 are presented in Table 2. Age was not correlated with either the total VHI-10 score or any individual subscale score (p > 0.05).

**Table 2:** Test-retest reliability of the Malay-VHI-10 scores assessed by ICC (n = 165).

	Mean ± SD	Mean ± SD	ICC	95% CI
VHI-10 scores	Test 1	Test 2		
Total	4.21 ± 5.15	3.23 ± 4.16	0.77	0.69–0.83
Functional	2.65 ± 2.78	1.89 ± 2.18	0.74	0.64–0.81
Physical	1.14 ±1.72	1.07 ± 1.57	0.78	0.70–0.84
Emotional	0.41 ± 1.13	0.27 ± 0.80	0.65	0.52–0.74

The Spearman correlation coefficient and Cronbach alpha for total VHI-10 score was 0.72 (p < 0.001) and 0.77 respectively showing good correlation and internal consistency. The ICCs ranged from 0.65 to 0.78 showing fair to good reliability and demonstrating the subscales to be reliable and stable.

### Phase II

Questionnaires were distributed to 6856 teachers, and 6039 (88.1%) returned the questionnaires. The respondents were primarily Malays, females, married, had tertiary education and in the age group of between 30 and 50 years (mean age in years; 95% CI: 42.18; 40.89, 43.46). A total of 10.4 (95% CI: 7.1, 14.9) % of teachers had a voice disorder (VHI-10 score > 11)(Table 3).

**Table 3:** Socio-demographic characteristics and prevalence of voice disorder among respondents.

Socio-demographic characteristics	Unweighted count (n)	^+^Weighted % (95%CI)
Gender:		
Male	973	13.2 (8.4, 20.1)
Female	5066	86.8 (79.9, 91.6)
Race		
Malays	4738	78.8 (63.8, 88.7)
Chinese	851	13.1(5.6, 27.6)
Indian	393	7.2 (5.1, 10.0)
Others	57	0.9 (0.5, 1.6)
Marital status*		
Single	645	10.6 (6.1, 17.9)
Married	5206	87.2 (82.7, 90.7)
Divorced/Widowed	133	2.1 (0.7, 5.8)
Age group (years)*		
≤ 29	580	9.7 (5.5, 16.6)
30–39	1761	29.6 (26.4, 33.1)
40–49	2445	40.3 (32.3, 48.9)
≥50	1231	20.4 (19.3, 21.4)
Education level*		
Diploma	264	3.8 (2.3, 6.3)
Degree	5044	84.4 (82.4, 86.3)
Master / PhD	676	11.7 (10.4, 13.2)
Voice disorder* (VHI-10 score > 11)		
Yes	554	10.4 (7.1, 14.9)
No	5124	89.6 (85.1, 92.9)

* Total less than 6039 as some data was missing

^+^ weight was counted based on the number of states, schools and teachers responded

Table 4 shows there is no difference in the proportions of males to females, age groups, education levels and years of teaching with voice disorder. Amplifier use, voice-related absenteeism and measures to minimise voice problems were significantly associated with the presence of voice disorder. A higher proportion of ethnic Chinese teachers were reported to have voice disorder (p = 0.048). There was a higher proportion of participants with a single and widowed/divorce status who had voice disorder compared to those who were married, however the finding was not statistically significant (p = 0.089).

**Table 4:** Association of socio-demographic characteristics and teaching characteristics with voice disorder.

Socio-demographic characteristics	Voice disorder		
	Yes	No	p value
	Unweighted count (^+^Weighted %)	Unweighted count (^+^Weighted %)	
Gender			
Male	81 (10.6)	851 (89.4)	0.861
Female	473 (10.1)	4273 (89.9)	
Race			
Malays	394 (9.4)	4055 (90.6)	0.048
Chinese	122 (16.2)	691 (83.8)	
Indian	31 (9.3)	334 (90.7)	
Others	7 (12.9)	44 (87.1)	
Marital status			
Single	76 (13.9)	535 (86.1)	0.089
Married	459 (9.9)	4439 (90.1)	
Divorced/Widowed	17 (13.6)	113 (86.4)	
Age group (years)			
≤ 29	64 (12.2)	485 (87.8)	0.413
30–39	171 (10.3)	1467 (89.7)	
40–49	229 (10.9)	2103 (89.1)	
≥ 50	90 (8.5)	1069 (91.5)	
Education levels			
Diploma	26 (11.6)	226 (88.4)	0.473
Degree	470 (10.6)	4293 (89.4)	
Master / PhD	57 (8.2)	586 (91.8)	
Years of teaching			
< 5	69 (13.1)	528 (86.9)	0.440
5–9.9	96 (10.5)	752 (89.5)	
10–14.9	93 (10.5)	889 (89.5)	
15–19.9	113 (10.6)	1061 (89.4)	
≥20	176 (9.3)	1838 (90.7)	
*Voice related absenteeism	224 (18.2)	326 (8.0)	0.001
*Amplifier use	76 (23.6)	473 (9.4)	0.013
*Measures to minimize voice problems	53 (20.2)	498 (9.9)	0.014

* column percent

^+^ weight was counted based on the number of states, schools and teachers responded

The mean VHI-10 score was 4.17 (95% CI: 3.86, 4.48). The means of medical leave (in days), total VHI-10, functional, physical and emotional scores for VHI10 were significantly higher among those with a voice disorder. The SF12v2-MCS scores measuring mental health-related quality of life were significantly lower; while depression, anxiety and stress scores were higher (but not statistically significant) among teachers who had voice disorder (Table 5).

**Table 5:** Association of weighted means (95% CI) of teaching hours, medical leave, health related quality of life, stress, anxiety and depression scores with voice disorder.

	Total	Voice Disorder	
	^+^Weighted mean		
Characteristics	(95% CI)	Yes	No
		^+^Weighted mean	^+^Weighted mean
		(95% CI)	(95% CI)
Age (years)	42.18 (40.89, 43.46)	41.72 (37.94, 45.49)	42.27 (41.33, 43.21)
Years of teaching	15.91(15.07, 16.75)	15.27 (12.10, 18.44)	15.89 (15.42, 16.54)
Hours of teaching/day	4.49 (4.23, 4.76)	4.73 (4.25, 5.20)	4.47 (4.23, 4.71)
Medical leave (days)*	2.00 (1.97, 2.03)	2.61 (2.30, 2.92)	1.88 (1.83, 1.94)
VHI-10 score(Total)*	4.17 (3.86, 4.48)	15.73 (14.79, 16.67)	2.83 (2.64, 3.02)
Functional VHI*	2.61 (2.46, 2.76)	7.94 (7.53, 8.36)	1.99 (1.80, 2.17)
Physical VHI*	1.11 (0.99, 1.22)	4.73 (4.29, 5.16)	0.68 (0.67, 0.69)
Emotional VHI*	0.47 (0.42, 0.52)	3.05 (2.36, 3.75)	0.17 (0.15, 0.19)
SF12-v2			
Physical Component Summary Scale	46.76 (42.92, 50.61)	45.38 (43.16, 47.59)	46.75 (42.79, 50.5)
Mental Component Summary Scale*	48.76 (47.35, 50.17)	44.07(42.91, 45.24)	49.30 (48.01, 50.59)
DASS21			
Stress	11.44 (5.19, 17.69)	16.31 (10.53, 22.10)	10.16 (6.39, 13.93)
Anxiety	9.94 (3.79, 16.09)	14.88 (8.45, 21.32)	8.68 (5.01, 12.35)
Depression	7.79 (1.37, 14.21)	12.31 (5.86, 18.76)	6.55 (2.65, 10.46)

*p<0.05, between groups of voice disorders (Yes vs No)

^+^ weight was counted based on the number of states, schools and teachers responded

Table 6 shows that the prevalence ratios (PR) for amplifier use, measures to minimise voice problems, voice-related absenteeism, stress, anxiety and depression scores were significantly higher among teachers with voice disorders. SF12v2-PCS and MCS scores were inversely associated with voice disorder in univariate analysis.

**Table 6:** Crude and adjusted prevalence ratios (PR) of determinants with voice disorder.

	Crude PR (95% CI)	^#^Adjusted PR (95% CI)
Gender:		
Male	1.00	-
Female	0.98 (0.66, 1.44)	
Race:		
Malays	1.00	1.00
Chinese	1.72 (0.98, 3.03)	1.33 (1.02,1.75)
Indian	0.99 (0.88, 1.11)	0.71 (0.66, 0.76)
Others	1.37 (0.85, 2.19)	1.06 (0.64, 1.75)
Marital status:		
Single	1.41 (0.92, 2.14)	1.39 (1.22, 1.60)
Married	1.00	1.00
Divorced/Widowed	1.38 (0.97, 1.97)	1.31 (1.10, 1.56)
Education level:		
Diploma	1.09 (0.43, 2.78)	-
Degree	1.00	
Master / PhD	0.78 (0.40, 1.49)	
Age group (years):		
≤ 29	1.44 (0.52, 4.01)	1.09 (0.47, 2.52)
30–39	1.22 (0.89, 1.65)	1.13 (0.94, 1.35)
40–49	1.29 (1.04, 1.59)	1.20(1.09, 1.32)
≥ 50	1.00	1.00
Years of teaching:		
< 5	1.41 (0.93, 2.14)	-
5–9.9	1.13 (0.44, 2.78)	
10–14.9	1.13 (0.93, 1.38)	
15–19.9	1.14 (0.88, 1.49)	
≥20	1.00	
Hours of teaching/day	1.00 (0.99, 1.00)	-
Amplifier use	2.51 (1.40, 4.50)	1.84 (0.89, 3.81)
Measures to minimize voice problems	2.04 (1.35, 3.09)	1.13 (0.58, 1.19)
Voice related absenteeism	2.28 (1.80, 2.87)	1.70 (1.33, 2.19)
SF-12v2:		
Physical Component Summary Scale	0.98 (0.95, 0.99)	0.98 (0.96, 0.99)
Mental Component Summary Scale	0.94 (0.93, 0.95)	0.97 (0.96, 0.98)
DASS 21:		
Stress	1.08 (1.04, 1.11)	1.00 (0.96, 1.06)
Anxiety	1.08 (1.04, 1.11)	1.04 (1.02, 1.06)
Depression	1.08 (1.04, 1.12)	1.02 (0.99, 1.04)

^#^ Adjusted for race, marital status, age group, years of teaching, amplifier use, measures to minimise voice problems, voice related absenteeism, SF12v2-PCS, SF12v2-MCS, depression, anxiety and stress scores

After adjusting for race, marital status, age group, years of teaching, amplifier use, measures to minimise voice problems, voice related absenteeism, SF12v2-PCS, SF12v2-MCS, depression, anxiety and stress scores; race, marital status, voice related absenteeism, SF12v2-PCS, SF12-v2-MCS and anxiety scores were significantly associated with voice disorder.

## Discussion

There were good internal consistency and reliability for the Malay-VHI-10, with high correlation and fair to good ICC scores between test-retest scores. These suggest that the questionnaire is reliable and responses obtained from this questionnaire were stable.

The correlation coefficient from our study (r = 0.72) was comparable with other VHI-10 versions translated in different languages such as Spanish (r = 0.85)[19], Hebrew (r = 0.92)[18] and Chinese (r = 0.84) [17]. With respect to the subscale scores from the repeated Malay version of VHI-10, the ICC in the emotional domain was the lowest but still within the range of good reliability. Emotions tend to vary and fluctuate over time due to other contributing factors, which may affect the reliability of this domain. Individuals may also be more familiar with their physical and functional symptoms, which are more prominent than emotional parameters. We conclude that the Malay-VHI-10 can serve as a tool in the evaluation of voice disorders among individuals or teachers who understand the Malay language.

The teachers who participated in the Phase II study were mostly Malays, females, married and had tertiary education. These characteristics corresponded well with teacher characteristics within the overall teaching profession in the public secondary schools of the country. Their mean VHI-10 score was higher than the normative value (2.83) of non-teachers [15] and 10.4% (95% CI: 7.1, 14.9) of them were found to have voice disorder. Although not explored in our study, teachers were documented to have higher risks for voice disorder compared to the general population [4,30]. The prevalence of voice disorder among teachers in this study using VHI-10 was considered low compared to studies by Roy et al, Sampaio et al and Sliwinska-Kowalska et al [27,30,31] with prevalence ranging between 20 and 30%. However, our study was comparable to some other studies [4,30,32] which reported the prevalence of about 10 to 12%. This variety in prevalence across studies may be due to disparity in the tools used or definition of voice disorder. Some studies also presented lifetime prevalence instead of point prevalence. Caution should be taken while comparing prevalence of voice disorder from different studies.

Contrary to other studies [4,9,27,33], we did not find female teachers to be predisposed for voice disorders. This could be confounded by the level of teaching as our teachers were secondary school teachers while studies elsewhere showed that kindergarten and elementary education teachers who were predominantly females had more voice symptoms than middle or high school teachers [7].

Compared to Malays, a greater proportion of ethnic Chinese teachers reported having voice disorders while ethnic Indian teachers were less likely to report having voice disorders. As all our teachers were proficient in the Malay language, misinterpretation of statements in Malay-VHI-10 was not an issue. However, perception of the impact of voice disorders may differ across different cultural backgrounds. This warrants further investigations, as there has been no concrete evidence on racial predisposition for voice disorders. Teachers who were single and divorced/widowed had a higher prevalence ratio for voice disorder. This could probably due to single parents having greater vocal demand at home, for instance if they were solely responsible for childcare. We postulate that single parents may also be at higher risk for high job family role strain and reduce levels of wellbeing, thus reported higher prevalence ratio of voice disorders. We think this is a new dimension on the issue at hand and further investigation is needed as this was not assessed in our study.

Usage of amplifiers and measures to minimise voice problems such as reducing class size or changing teaching subjects were significantly associated with voice disorder at the univariate level, similarly reported by Chen et al [11]. Use of amplifier may reduce the need for teachers to project their voice above background noise, as well as overall vocal load and has been accepted as a clinical utility to reduce voice disorders among teachers [34–36]. Having a smaller class size may be another way of overcoming the need to raise one’s voice [9]. However, such measures are not under the teachers’ control as the school management normally decides class size. Our findings became non-significant after adjusting for confounders.

We found more voice-related absenteeism among teachers with voice disorders, as reported by Behlau et al. [4]. Voice disorders may force teachers to increase the rates of absenteeism, which will affect teaching quality and create discontinuities in the curriculum with detrimental effects on student learning [4,7].

Health-related quality of life score in the aspects of PCS and MCS were inversely associated with voice disorder; while those with voice disorder were more likely to have anxiety. Although these associations were statistically significant, they did not appear to be clinically significant as the magnitude of the measure of association was small. We did not explore the reasons for the lower proportions of psychological distress or anxiety among our teachers compared to the teachers in other studies as education systems and teaching environment between countries differ. On the other hand, these results had some clinical relevance as reduction of communicative and social ability; and emotional instability for teachers with voice disorders may result in anxiety and deterioration of health-related quality of life. This was shown in two studies in Europe and Egypt, where teachers with voice disorders presented a higher level of psychological distress (p < 0.001) [37] or anxiety [38] compared to teachers without voice problems.

There are several limitations which warrant consideration while interpreting the results. Firstly, this is a cross-sectional study in which causality cannot be established. Secondly, as this study only included secondary school teachers, the magnitude of voice disorder may not be generalised to primary and preschool teachers. Finally, the representation of male and non-Malay (ethnic Chinese and Indian) teachers in this study population was lower compared to the country’s population composition. Therefore, these results should be interpreted with caution.

This paper only reported the data of four states, however we feel additional data from two other states will not alter the results and conclusion of the study. This is because the two other states have similar racial composition and our study has adequate power. To the best of our knowledge, this is the first published study on voice disorder among Malaysian teachers. The sound sampling method which included both urban and rural schools, and large sample size provided sufficient power for the study.

Although the prevalence of voice disorders in our study is relatively low, voice disorder impacts on the teachers’ quality of life and mental health that will in turn affect their teaching performance. The use of amplifiers should be encouraged to reduced stress on the voice, either through sponsorship or subsidies by the Ministry of Education as the employer. In addition, it is the duty of the employer to consider voice disorders as a potential occupational risk for teachers.

Although the prevalence of voice disorder found among our participants was not high, we would still like to suggest that preventive programs for voice disorders to be implemented during teacher training and be reinforced throughout their career as they place heavy demand on their voice as the tool of teaching. Potential preventive strategies, such as voice screening, vocal health education and voice training could possibly be explored in the future, not just for its effect on voice disorders but to reduce the possible stress that comes with voice disorders. Occupational safety and health policies on occupational voice disorders should be established and reviewed regularly in accordance with emerging evidence.

In conclusion, we found the Malay-VHI-10 is valid and reliable for assessment of voice disorder among individuals who use the Malay language. The prevalence of voice disorder among secondary school teachers in our study population was 10.4%. Determinants such as race and marital status were associated with voice disorder. Voice disorder was associated with increased absenteeism, marginally associated with reduced health-related quality of life (PCS and MCS) as well as increased anxiety of teachers.

We recommend that voice disorders be recognized as an occupational disorder among teachers in Malaysia and appropriate educational and preventive measures be taken. A prospective study which includes primary and preschool teachers of more non-Malays should be carried out.
